# A survey of schoolchildren's exposure to secondhand smoke in Malaysia

**DOI:** 10.1186/1471-2458-11-634

**Published:** 2011-08-08

**Authors:** Emilia Zainal Abidin, Sean Semple, Affandi Omar, Hejar A Rahman, Stephen W Turner, Jon G Ayres

**Affiliations:** 1 Scottish Centre for Indoor Air, Environmental & Occupational Medicine, Population Health, Division of Applied Health Sciences, School of Medicine and Dentistry, Polwarth Building, Foresterhill, University of Aberdeen, AB25 2ZD Aberdeen, UK; 2 Biochemistry Unit, Specialised Diagnostic Unit, Institute of Medical Research, Jalan Pahang, 50588 Kuala Lumpur, Malaysia; 3 Department of Community Health, Faculty of Medicine and Health Science, Universiti Putra Malaysia, 43400 Serdang, Selangor, Malaysia; 4 Child Health, University of Aberdeen, Royal Aberdeen Children's Hospital, Aberdeen AB25 2ZG Scotland UK; 5 Institute of Occupational and Environmental Medicine, University of Birmingham, Edgbaston Birmingham, B15 2TT, UK

**Keywords:** Secondhand smoke, salivary cotinine, schoolchildren, self-reported smoke exposure, smoke-free legislation, enzyme-immunoassay method

## Abstract

**Background:**

There is a lack of data describing the exposure of Malaysian schoolchildren to Secondhand Smoke (SHS). The aim of this study is to identify factors influencing schoolchildren's exposures to SHS in Malaysia.

**Method:**

This cross-sectional study was carried out to measure salivary cotinine concentrations among 1064 schoolchildren (10-11 years) attending 24 schools in Malaysia following recent partial smoke-free restrictions. Parents completed questionnaires and schoolchildren provided saliva samples for cotinine assay.

**Results:**

The geometric mean (GM) salivary cotinine concentrations for 947 non-smoking schoolchildren stratified by household residents' smoking behaviour were: for children living with non-smoking parents 0.32 ng/ml (95% CI 0.28-0.37) (n = 446); for children living with a smoker father 0.65 ng/ml (95% CI 0.57-0.72) (n = 432); for children living with two smoking parents 1.12 ng/ml (95% CI 0.29-4.40) (n = 3); for children who live with an extended family member who smokes 0.62 ng/ml (95% CI 0.42-0.89) (n = 33) and for children living with two smokers (father and extended family member) 0.71 ng/ml (95% CI 0.40-0.97) (n = 44). Parental-reported SHS exposures showed poor agreement with children's self-reported SHS exposures. Multiple linear regression demonstrated that cotinine levels were positively associated with living with one or more smokers, urban residence, occupation of father (Armed forces), parental-reported exposure to SHS and education of the father (Diploma/Technical certificate).

**Conclusions:**

This is the first study to characterise exposures to SHS using salivary cotinine concentrations among schoolchildren in Malaysia and also the first study documenting SHS exposure using salivary cotinine as a biomarker in a South-East Asian population of schoolchildren. Compared to other populations of similarly aged schoolchildren, Malaysian children have higher salivary cotinine concentrations. The partial nature of smoke-free restrictions in Malaysia is likely to contribute to these findings. Enforcement of existing legislation to reduce exposure in public place settings and interventions to reduce exposure at home, especially to implement effective home smoking restriction practices are required.

## Background

There is established evidence linking Secondhand Smoke (SHS) exposures to poor health outcomes [1-4]. Among adults, exposures to SHS have been linked to outcomes such as chronic obstructive pulmonary disease [1], deficits in lung function [2], increased risk of adult-onset asthma [3], and increased risk of lung cancer [4]. SHS exposures among children have been linked with outcomes such as asthma [5], exacerbations of respiratory ill-health [6], otitis media [7], sudden infant death syndrome [8] and poor cognitive development [9].

In Malaysia, the prevalence of smoking in adults has been estimated at 25% [10]. This figure was derived from a cross-sectional study of more than 17,000 Malaysian respondents in 2004 (matching the ethnic distribution of approximately 56% of Malays, 21% Chinese and 11% Indians) and is similar to that obtained for other countries including Scotland [11]. However, unlike Scotland and the rest of the United Kingdom (UK), the Malaysian data is highly skewed by gender with a high smoking prevalence among males (47%) with significantly less among females (3%). As in many countries in the Western Pacific Region, Taiwan have a similar skewed distribution of smokers and a national survey in Taiwan reported that half of all women and children were exposed to SHS at home [12]. The percentage of homes in Malaysia where SHS exposure occurs may be similar to that found in Taiwan.

Most of the studies on tobacco smoke exposure among children identified parental smoking as the main contributor to SHS exposure at home, and in particular maternal smoking. An estimated 31.7% of children report living with a smoker in Scotland and with a similar figure reported in Wales [13,14]. Elsewhere in the European Union, parental smoking prevalence in Greece was found to be the highest, estimated at 44% [15]. In the UK, children's exposure to SHS has been declining in recent years. The Geometric Mean (GM) of salivary cotinine as a biomarker of SHS exposures reduced from 0.6 ng/ml in 1996 to approximately 0.2 ng/ml in 2006 [16].

Article 8 in the World Health Organisation's (WHO) Framework Convention of Tobacco Control (FCTC) aims to reduce children's exposure to SHS. Ratification of the FCTC leads to the introduction of Smoke-Free Legislation (SFL) among its member countries with SFL being introduced in many countries in the last decade. The extent of SFL varies from country to country: some countries such as Scotland and Ireland have introduced complete smoke-free environments in all enclosed public spaces with very few exemptions. In Malaysia, signing of the WHO's FCTC was performed in 2003 with ratification in 2005. Legislation partially restricting smoking in public places was introduced in 2004 under the Control of Tobacco Products Regulations under the Food Act 1983 [17]. Smoking restrictions covered five types of locations in 2007 including government offices, health facilities, educational facilities, public transport and air-conditioned venues with further changes in 2008 to include a total of 19 public-space venue types as smoke-free [18]. The designated public areas where smoking restrictions are in place now include air-conditioned restaurants, public transport, internet cafes and shopping complexes. However, smoking is still allowed in some enclosed public spaces and many outdoor areas such as semi-enclosed eating establishments.

We are not aware of any literature describing the SHS exposure of Malaysian children other than a small number of publications on Malaysian adolescent active smoking [19-22]. Given the relatively low prevalence of maternal smoking in Malaysia compared to other countries, Malaysian children's SHS exposure may be lower than in other countries. One study of Malaysian university students measured urinary cotinine levels in 959 university students aged 18 to 24 years old and found a GM value among non-smokers of 4.6 ng/ml [23].

To our knowledge this is the first study of SHS exposure among schoolchildren in Malaysia and also the first study documenting SHS exposure using salivary cotinine as a biomarker in a South-East Asian population of schoolchildren.

## Methods

This study is part of a larger cross-sectional survey looking at exposure to indoor air pollution and the effect on respiratory health in schoolchildren in Malaysia. Among the associated factors studied were SHS exposures from tobacco.

Data were collected during April to September 2009. The study was performed in two different areas of Malaysia, urban Kuala Lumpur and three rural districts in Negeri Sembilan. While not a strict representative sample of the Malaysian population, the two identified areas are broadly representative of Malaysian urban and rural communities. Kuala Lumpur was chosen because it represents the largest urban area in Malaysia with the city population making up approximately one quarter of the current total Malaysian population. Negeri Sembilan was selected to represent more rural or semi-rural Malaysian areas. It is located about 64 km south of Kuala Lumpur and much of the economic activity is centred around agriculture.

### Ethical approval and permissions

Ethical approval was obtained from the College of Life Sciences and Medicine's Ethical Review Board, University of Aberdeen. Permission was also obtained from the Economic Planning Unit at the Prime Minister's Department in Malaysia. Approvals from respective schools were requested before the data collection commenced. Upon being granted permission to enter schools, arrangements were made with the school administrative board to ensure research was performed during a convenient time period. Written informed consent was obtained from parents.

### Selection of schools and recruitment of participants

The children who took part in this survey were recruited from National (government funded) Schools listed in the Education Management Information System directory made available in the website of the Ministry of Education Malaysia. Primary educations in Malaysia consist of six years of education, Year 1 to Year 6, which begins at the age of 7 and ends at age 12. As only national schools were selected, the selections were not representative of the general school distribution in Malaysia with less representation of children from Chinese and Indian backgrounds. Forty schools in urban Kuala Lumpur and three rural districts in Negeri Sembilan namely the districts of Jempol, Jelebu and Kuala Pilah were contacted. In total 36 schools responded and gave their permission to be included in the study. In order to cover as large a geographical area of Kuala Lumpur and rural districts as possible, where responding schools were less than 2 km apart, only one was selected to participate. This selection was performed randomly. This resulted in a total of 24 schools selected to be included in the salivary cotinine part of the study. From each school, a minimum of two classrooms from the year 4 and year 5 groups were randomly chosen to be included in the study. All children in selected classes of each participating school were invited to take part in the study.

### Questionnaires

Questionnaires were completed by a parent or the guardian of the child who agreed to participate. Completed questionnaires were returned to the team of researchers within 3 days and prior to the collection of saliva samples which took place during school hours. The questionnaire collected demographic information and details of household smoking behaviours. Children were grouped into three categories by total household earnings using the classification given by the Department of Statistics, Malaysia [24]. The questionnaire was developed in English before being translated by a native Malay speaker. Part of the questions on smoking habits of parents were taken from previously validated questionnaire. All children were able to converse in Malay language. Questionnaires with missing information on gender, family structure, ethnicity, home smoking restrictions and family income were excluded from further statistical analysis.

Schoolchildren who took part were given a 48-hour location-activity diary to be completed. This diary enabled collection of location and activity data with a resolution of 30 minutes. Data collected included information on the activity and whether this was at home, in school or in another indoor or outdoor location. The diary also includes a column which enquired about the presence of SHS in the proximity of their location for every 30-minute interval. For this paper, only the information gathered from the total hours of SHS exposures for 48 hours was used for analysis to enable comparison between child self-report and parental-report of their child's SHS exposures.

### Saliva sample collection

Saliva was collected from each child using a sterile dental cotton roll (Salivettes; Sarstedt, Germany) and based on a protocol similar to that used in the CHETS (Changes in child exposure to environmental tobacco smoke) and BHETSE (Bar workers' Health and Environmental Tobacco Smoke Exposure) studies in Scotland [13,25]. Since this is a school-based study, sample collection was performed in schools in the presence of one of the research team [EZA]. Children were requested to place the cotton roll in their mouth for two minutes in order to produce an adequate volume of saliva for later analysis. Samples collected were kept in an ice box within 2 minutes of collection before being transported to the laboratory within 2 hours and being stored in a -20°C freezer pending analysis.

### Cotinine analysis

All analysis was performed at the Specialised Diagnostic Unit, Biochemistry Laboratory at Institute of Medical Research in Kuala Lumpur, Malaysia. The concentration of salivary cotinine was determined using an Enzyme Immunoassay kit (EIA Cozart Bio-Science Ltd.). The concentrations of salivary cotinine were obtained by extrapolating optical density values from standard curves graphs obtained from analysis of standard concentrations from the standard cotinine calibrator set manufactured by Cozart. Cotinine concentrations were expressed in ng/ml. The analytical limit of detection for this method of analysis was derived from using a value which is three times the standard deviation of the blank standard concentration. The limit of detection is 0.1 ng/ml. For comparison purposes in our analysis we identified samples that had cotinine concentrations of 1.7 ng/ml and above. This value has been reported to be associated with impairment of endothelial function in children [26]. We also classified samples in relation to cotinine concentrations of 3 ng/ml and higher, a value representing the average salivary cotinine concentrations measured in highly exposed non-smoking bar workers prior to the implementation of smoke-free legislation in Scotland [25].

### Statistical analysis

Data were entered and analysed using a commercial statistical programme (PASW Statistics version 18). Group data were explored and due to the skewness of cotinine distribution arising from a large number of lower cotinine concentration values, group cotinine concentration is expressed in GM. Samples with cotinine concentration of less than a limit of detection (0.1 ng/ml) were assigned an imputed value randomly sampled from the left tail of a truncated log normal distribution [13]. The differences of cotinine concentrations between two different groups were tested using the independent *t*-test and the differences between more than two groups were tested using ANOVA test and were further tested using post-hoc tests. Categorical variables were tested using the chi-square test with significance assigned where the *p*-value is less than 0.05. Variables found to be significant in the univariate analysis were then included in a multiple linear regression to identify predictors of the distribution of cotinine concentrations.

## Results

### Response rates

Study information packs including questionnaires were distributed to 2775 children in 16 urban schools and 8 rural schools.

A total of 2018 (72.7% response rate) study packs were returned with 1785 of these having completed questionnaires and permission forms. However, from that figure, 1254 of the children had additional parental permission to participate in the saliva collection element of this study. Due to logistical difficulties and classroom absence on the day of data collection we managed to collect samples from a total of 1172 (93%) of children for whom we had consent to collect saliva. From this set 1064 (91%) saliva samples were successfully analysed by the laboratory to yield cotinine concentrations.

### Characteristics of participants

The characteristics of the 1785 participating children are provided in Table 1. Table 1 also provides comparison of the demographics of the children from whom we were able to collect and analyse saliva in the total study cohort and in those children who opted out of the study. There were no differences between the children who opted out of the study/or did not provide a sample/or have insufficient saliva samples for laboratory analysis (n = 721) and the children for whom we had a valid saliva sample from (n = 1064) except for the number of children living in non-smoking households and classified as coming from a family with a low income.

**Table 1:** Description of the selected sample population of Malaysian schoolchildren

Characteristics	Number opted out of study (n = 721)^I, a^	Number consented to salivary cotinine study (n=1064)^II^	Total^III^ (n = 1785)
**Mean age (years ± SD)**	10.7 ± 0.6	10.7 ± 0.6	10.7 ± 0.6
**Gender**			
*Boys*	316 (43.8)	448 (42.1)	764 (42.8)
*Girls*	380 (52.7)	605 (56.7)	985 (55.2)
*Missing*	25 (3.5)	11 (1.0)	36 (2.0)
**Family structure**			
*Both parents*	651 (90.3)	997 (93.7)	1648 (92.3)
*Single parent*	21 (2.9)	27 (2.6)	48 (2.7)
*Others*	30 (4.1)	40 (3.8)	70 (3.9)
*Missing*	19 (2.6)	-	19 (1.1)
**Ethnicity**			
*Malay*	635 (88.1)	958 (90.0)	1593 (89.2)
*Others*	67 (9.3)	106 (10.0)	173 (9.7)
*Missing*	19 (2.6)	-	19 (1.1)
**Location**			
*Urban*	435 (60.3)	688 (64.7)	1123 (62.9)
*Rural*	286 (39.7)	376 (35.3)	662 (37.1)
**Home smoking characteristics^++^**			
*Non-smoking households*	377 (52.3)	446 (41.9)	823 (46.1)
*Smoking households*	290 (40.2)	501 (47.1)	791 (44.3)
*Missing *	54 (7.5)	117 (11.0)	171 (9.6)
**Family income^++^**			
*Low*	319 (44.2)	529 (49.7)	847 (47.6)
*Medium*	274 (38.0)	326 (30.6)	601 (33.7)
*High*	88 (12.2)	144 (13.5)	232 (13.0)
*Missing*	40 (5.5)	65 (6.1)	105 (5.9)

Values are in numbers (percentages)

SD: Standard Deviation

Chi-square test performed between column ^I ^and ^II ^(excluding missing values)

^++^: Chi-square outcome for column ^I ^and ^II ^was *p *< 0.05 for home smoking characteristics and family income group

** The 3 categories in the variable Family income was derived using cut-off points obtained from the recent data of Economic Planning Unit, Prime Minister Department Malaysia. The average income is RM3686. Family income lower than RM3000 per month is categorised as low income, RM3000 to RM5000 is categorised as middle income and income of more than RM5000 is categorised as high income group

**^a ^**Column I represents 721 children which includes 531 children who opted out of the study, 82 children whose saliva samples could not be collected and108 children who did not provide sufficient saliva sample for laboratory analysis

### Distribution of SHS exposure

Salivary cotinine concentrations ranged from less than LOD to 12 ng/ml. Previous work has used a cut-off point of more than 15 ng/ml of salivary cotinine levels as evidence of active smoking [12] and so using this definition all study participants were classified as non-smokers.

The overall GM of salivary cotinine for the schoolchildren in this study is 0.46 ng/ml (95%CI 0.42-0.50) with a median of 0.72 ng/ml. Schoolchildren were categorised into two groups to discern whether they were living in non-smoking or smoking homes. From a total of 1064, the number of schoolchildren that could be included into further analysis is reduced to 947 due to missing information on household smoking characteristics.

**Figure 1:**
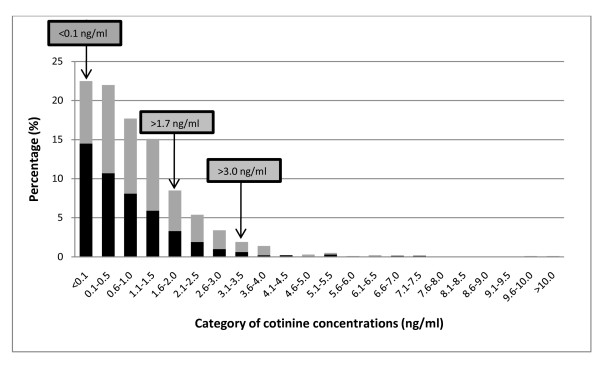
**Distribution of salivary cotinine concentrations among the Malaysian schoolchildren (n = 947)**. Grey bar represents smoking homes and black bar represents non-smoking homes.

Figure 1 describes the distribution of cotinine concentrations for schoolchildren in non-smoking and smoking homes. Approximately 22.7% of the Malaysian schoolchildren had cotinine concentrations below the limit of detection (0.1 ng/ml). The percentage of children who had salivary cotinine concentrations above 1.7 ng/ml [26] and 3.0 ng/ml [25] is 18.0% and 5.0% respectively.

### Household smoking and children's salivary cotinine concentrations

Table 2 provides details of children's salivary cotinine concentrations across different household smoking arrangements. From the 947 children with complete information on smoking behaviours at home, 52.9% of them lived in a household with at least one smoker. Smokers in households are not restricted to father and a small number of mother figures, but also included uncle (n = 24) and brother (n = 42). The number of smokers in the households was between the range of 1 to a maximum of 2.

**Table 2:** Malaysian children's geometric mean salivary cotinine concentration categorised by adult smoking within the home

Smoke exposure at home (number of smokers at home)	GM cotinine		Total GM cotinine^III^ (95% CI) ng/ml	Total (%)
			
	Urban (n)^I^	Rural (n)^II^		
Neither parent smokes (0)	0.38 (322)*	0.22 (124)*	0.32 (0.28-0.37)^++^	446 (47.1)
Father only smokes (1)	0.80 (240)*	0.50 (192)*	0.65 (0.57-0.72)^++^	432 (45.6)
Father and mother smokes (2)	1.54 (2)	0.60 (1)	1.12 (0.29-4.40)	3 (0.3)
Extended family member smokes (1)	0.73 (23)	0.42 (10)	0.62 (0.42-0.89)	33 (3.5)
Father and extended family member smokes (2)	0.86 (13)	0.63 (20)	0.71 (0.40-0.97)^++^	33 (3.5)
**Total**	0.54 (600)	0.38 (347)	0.47 (0.42-0.50)	947

* *t*-test performed between categories in column ^I ^and ^II^, *p *< 0.05

ANOVA test performed for column ^III ^and *F *= 9.737; *p*-value < 0.0001

^++ ^*LSD *Post-hoc test was performed for column III and significant differences were found between the group neither parents smokes with all the other group of smoke exposure at home except for extended family member smokes (category where "Father and mother smokes" was excluded from analysis)

The presence of one or more adult smokers in the home was associated with higher GM cotinine concentrations when compared to children living in non-smoking households (0.32 v 0.65 ng/ml, *p *< 0.0001). Among children living with non-smoking parents, the GM salivary cotinine concentration was 0.32 ng/ml (95%CI 0.28 to 0.37). Children who live in households where only the father smokes had GM cotinine concentrations of 0.65 ng/ml (95% CI 0.57-0.72). Levels of cotinine concentrations were higher when more than one family member smokes, with the highest concentrations in the three children who had both a smoking mother and father with cotinine concentrations of 1.12 ng/ml (95% CI 0.29-4.40). Schoolchildren who lived with an extended family member who was a smoker had cotinine concentrations of 0.62 ng/ml (95% CI 0.42-0.89) and children living with a father and a family member who smoked had cotinine concentrations of 0.71 ng/ml (95% CI 0.40-0.97).

### Relationship of salivary cotinine concentrations with socio-demographics and household characteristics

Table 3 shows GM cotinine concentrations according to location of residence, gender of child, education and occupation of father, family income, presence of a smoking adult resident, location of the child's sleeping area, the use of exhaust fan and air-conditioner in home. Children from urban households had significantly higher GM cotinine concentration when compared to children living in rural households (0.54 v 0.36 ng/ml, *p *< 0.0001). Among all children, GM cotinine concentrations were found to be similar among boys and girls (0.48 v 0.46 ng/ml, *p *= 0.576) and across different income groups low (0.46 ng/ml), middle (0.49 ng/ml) and high (0.39 ng/ml). For children who lived with one or more adult smokers at home, their salivary cotinine concentrations were not influenced by their sleeping arrangements.

**Table 3:** GM cotinine concentrations (GM cot (ng/ml)) and univariate analyses by different socio-demographic characteristics in Malaysian schoolchildren

Socio-demographic and household characteristics	Urban (n)^I^		Rural (n)^II^		Total (n)^III^	
	
	GM cot	*p*-value	GM cot	*p*-value	GM cot	*p*-value
**Geometric mean**	0.54 (688)*		0.36 (376)*	*t *= 4.069		*p *< 0.0001
**Gender^T ^**						
*Boy*	0.58 (284)	*t *= 1.144	0.35 (164)	*t *= -0.454	0.48 (448)	*t *= 0.559
*Girl*	0.51 (396)	*p *= 0.253	0.37 (209)	*p *= 0.650	0.46 (605)	*p *= 0.576
**Education of father^++a ^**						
*Middle school*	0.81 (29)	*F *= 3.593	0.36 (26)	*F *= 2.909	0.57 (159)	*F *= 3.328
*High school*	0.59 (399)*	*p *= 0.013	0.35 (245)	*p *= 0.035	0.49 (590)	*p *= 0.019
*Diploma/Technical cert*.	0.54 (81)		0.60 (45)*		0.58 (117)	
*College/University*	0.38 (129)*		0.25 (44)*		0.35 (159)*	
**Occupation of father^++b ^**						
*Manual non-skilled*	0.46 (180)	*F *= 10.926	0.40 (156)	*F *= 1.948	0.45 (280)	*F *= 9.445
*Skilled worker*	0.45 (101)	*p *< 0.0001	0.45 (64)	*p *= 0.122	0.44 (149)	*p *< 0.0001
*Armed forces*	0.91 (179)*		0.25 (30)		0.77 (198)*	
*Managerial/Professional *	0.41 (180)*		0.29 (79)		0.37 (242)	
**Family income^++^**						
*Low *	0.55 (262)	*F *= 1.819	0.38 (209)	*F *= 0.140	0.46 (528)	*F *= 1.316
*Middle *	0.57 (225)	*p *= 0.163	0.35 (83)	*p *= 0.869	0.49 (327)	*p *= 0.269
*High *	0.41 (96)		0.36 (38)		0.39 (144)	
**SHS in household^T ^**						
*Non-smoking*	0.38 (322)*	*t *= -6.23	0.22 (124)*	*t *= -5.323	0.32 (446)*	*t *= -7.329
*Smoking*	0.80 (278)*	*p *< 0.0001	0.51 (223)*	*p *< 0.0001	0.65 (501)*	*p *< 0.0001
**Child's sleeping area^++c^**						
*Own room/share siblings*	0.89 (207)	*F *= 1.789	0.55 (162)	*F *= 0.543	0.72 (369)	*F *= 2.724
*Living room*	0.60 (52)	*p *= 0.169	0.84 (32)	*p *= 0.582	0.54 (84)	*p *= 0.067
*Share with parents/adults*	0.49 (18)		0.79 (28)		0.40 (46)	
**Use of air-conditioner^++d ^**						
*None*	0.59 (462)*	*F *= 4.109	0.38 (295)	*F *= 0.807	0.51 (702)*	*F *= 3.804
*Living room*	0.35 (42)	*p *= 0.007	0.21 (11)	*p *= 0.490	0.34 (49)	*p *= 0.010
*Child's sleeping area*	0.36 (83)		0.28 (25)		0.32 (101)*	
**The use of exhaust system^T ^**						
*No*	0.58 (508)*	*t *= 2.485	0.38 (314)	*t *= 1.511	0.50 (756)*	*t *= 2.699
*Yes*	0.41 (146)*	*p *= 0.013	0.28 (55)	*p *= 0.132	0.36 (184)*	*p *= 0.007
**Smoking restriction in homes^++e^**						
*Total*	0.51 (437)	*F *= 2.150	0.35 (233)*	*F *= 2.333	0.45 (667)*	*F *= 3.464
*Partial*	0.65 (139)	*p = *0.117	0.38 (94)	*p = *0.099	0.52 (230)	*p = *0.032
*None*	0.75 (33)		0.71 (19)*		0.74 (52)*	

* Significant differences when *p *< 0.05

++ ANOVA test performed with *LSD *post-hoc test when *F *value have *p *< 0.05

**^T ^***t*-test performed

^a ^For education of father in column ^I^, GM between the group high school and college/university were significantly different

^b ^For occupation of father in column ^I^, GM between the groups armed forces and Managerial/Professional were significantly different

^c ^For children who live in smoking home only

^d ^For the use of air-conditioner in column ^I^, GM for all the groups were significantly different from None

^a ^For education of father in column ^II^, GM between the group Diploma/Certificate were significantly different from college/university level

^e ^For smoking restriction in homes in column ^II^, GM between total smoking restriction and no smoking restriction group were significantly different

**^a ^**For education of father in column ^III^, GM for all groups were significantly different when compared to college/university level

**^b ^**For occupation of father in column ^III^, GM for all groups were significantly different when compared to armed forces category

**^c ^**For child's sleeping area in column **^III ^**, GM between own room/share with siblings and share with parents/adults were significantly different

**^d ^**For the use of air-conditioner in column **^III ^**, GM between none and child's sleeping area were significantly different

^e ^For smoking restriction in homes in column ^II^, GM between total smoking restriction and no smoking restriction were significantly different

The education and occupation of the father, the use of air-conditioner and exhaust fans were all found to be significantly associated with the GM cotinine concentration in univariate analysis. Having a father with a Diploma/Technical certificate as opposed to a College/University level education (0.58 v 0.35 ng/ml, *p *= 0.0019) and who worked in the Armed forces as opposed to those who were Managers/Professionals was positively associated with GM cotinine concentrations (0.77 v 0.35 ng/ml, *p *< 0.0001). The use of air-conditioning in the child's sleeping area was also associated with a significantly lower GM cotinine concentration compared to those who did not use air-conditioners (0.32 v 0.51 ng/ml, *p *= 0.010). Finally, the use of mechanical ventilation such as an exhaust fan in the home was associated with lower GM cotinine concentrations when compared to homes without the additional exhaust fan (0.36 v 0.50 ng/ml, *p *= 0.007).

Salivary cotinine concentrations were categorised into three groups of home smoking restrictions being practised in the households of the schoolchildren. The three categories were: 1) smoking not allowed (total ban), 2) smoking allowed with exceptions (limited area) and 3) smoking allowed without exceptions (no restriction). A significant difference in cotinine concentrations was shown between children who lived in households with total or no smoking restrictions (0.45 v 0.74 ng/ml, *p *= 0.032).

### Parental perception and children's self-report of SHS exposures

In Table 4, parental report of their child's exposure hours to SHS was compared with the child's self-report of SHS exposure as recorded in the 48-hour activity diary. From 1064, a total of 959 data were available from the parents and 755 complete data were available from the children's diaries. Only 695 overlapping data were available for comparison. Statistical analyses of data from children who did not complete the diaries or had missing questionnaire information with those who had complete diary and questionnaire data were performed. It was found that there was no difference in the prevalence of adult resident smoking between these two groups.

**Table 4:** Parental perception of their child's SHS exposures daily in comparison with SHS exposures reported by their child

Exposure hours to SHS daily	Parental-reported exposure			GM cotinine (n)^~^
		
	None	1 to 3 hours	> 3 hours	
**Self-reported exposure**				
*None *	142 (61.7^+^%)	80 (34.8%)	8 (3.5%)	0.37^~ ^(230)
*1 to 3 hours*	174 (44.2%)	184 (46.7%)	36 (9.1%)	0.51^~ ^(394)
*> 3 hours*	17 (23.9%)	42 (59.2%)	12 (16.9%)	0.83^~ ^(71)
**GM cot (n/%)^++^**	0.38^++ ^(333/47.9%)	0.59^++ ^(306/44.0%)	0.69^++ ^(56/8.1%)	695

Chi-square test: 41.088 df: 4, *p *< 0.0001

^+ ^Percentages in bracket relate to the total of the self-reported exposure row.

^++ ^ANOVA test was performed with *LSD *post-hoc test and significant differences were observed between None with 1 to 3 and > 3 hours: *F *= 8.537, *p *< 0.0001

^~ ^ANOVA test was performed with *LSD *post-hoc test and significant differences were found between all three groups: *F *= 8.613, *p *< 0.0001

GM cotinine concentrations distributed according to parental-reported SHS exposures were significantly different across all three categories of exposure hours. Similarly, self-reported SHS exposures were too found to be significantly different between the three groups. Cross-tabulation was performed and significant differences in the distribution of frequency between the parental-reported and self-reported exposures of SHS daily were observed from the Chi-square test. Of children who reported no SHS exposure (n = 230), approximately 38% (n = 88) of their parents reported that their child had some SHS exposure. Children were less likely to agree with parents who reported their child having zero SHS exposure. For the 333 parents who reported their children having no SHS exposure, some 69% (n = 191) of these children recorded at least one 30-min exposure in the previous two days.

### Predictor of salivary cotinine concentrations

Results for the multiple linear regressions are provided in Table 5. Log salivary cotinine concentration was used as the dependent variable. In the model, it was found that children living in urban residential areas have higher predicted salivary cotinine levels than rural children. As in the univariate analysis, cotinine concentrations were higher for children living with one or more smokers, with the category of Father only smokes contributing the most towards the cotinine concentrations compared to children living with no smokers at home. Children whose father who worked in the Armed forces had higher cotinine concentrations when compared to the reference group (Managerial/Professional). It was also found children with fathers who had Diploma or Technical certificate education have higher salivary cotinine concentrations compared to children of fathers who had higher educational attainment. Parental-reported exposure hours to SHS of children's were higher among children categorised with 1 to 3 hours of exposures when compared to children categorised without exposure. Children who reported use of air-conditioning in the home had lower cotinine concentrations when compared to children who did not.

**Table 5:** Multiple linear regression coefficients

Variable in the model	Coefficients			*p*-value
		
	Unstd. B	Std. Error	Std. Beta	
**Location**				
*Urban*	0.44	0.11	0.14	<0.0001*
*Rural (Ref)*	0	0	0	
**Smoker**				
*Father only smoker *	0.44	0.12	0.15	<0.0001*
*Father and Family smoker*	0.78	0.30	0.09	0.010*
*Neither Parents smoke (Ref)*	0	0	0	
**Occupation of Father**				
*Armed Forces*	0.55	0.12	0.16	<0.0001*
*Manager/Professional (Ref)*	0	0	0	
**Education of Father**				
*Diploma/Technical cert*.	0.35	0.15	0.08	0.021*
*Degree/College (Ref)*	0	0	0	
**Parental-reported exposure hours to SHS**				
*1 to 3*	0.44	0.12	0.15	<0.0001*
*None (Ref)*	0	0	0	
**Air-conditioning in home**				
*Living room*	-0.72	0.24	-0.11	0.002*
*Child's sleeping area*	-0.39	0.16	-0.08	0.017*
*None (Ref)*	0	0	0	
**Constant**	-1.56	0.12		<0.0001*

Adjusted *R^2 ^*= 0.122; Ref: Reference category Unstd: Unstandardised Std.: Standardised

## Discussion

This study found that 52.9% of Malaysian schoolchildren were exposed to SHS at home, a much higher figure compared to studies in the UK [13-15]. Data from a previous study in Negeri Sembilan in Malaysia indicated that adolescents were exposed to parental smoking in 40% of homes [21]. In another study on smoking among young women in a private educational institution in Kuala Lumpur, Malaysia smoking among fathers was estimated at 50.9% [19], a value similar to that found in this study. We have compared our results to data from a study of salivary cotinine levels of similarly aged schoolchildren in Scotland [13]. We have compared our data to that available in Scotland because of the direct comparability of the age group sampled and the similar cross-sectional study design. Data from Scotland was gathered around the time SFL was implemented there in 2006 during a period of societal changes in attitudes towards SHS. Our data was collected during changes to Malaysian SFL and represents the exposure of children to SHS within a system of partial restrictions.

The overall GM value (0.46 ng/ml) of salivary cotinine from our sample of over 1000 Malaysian school children can be compared with other studies of children internationally. Delpisheh and colleagues measured salivary cotinine among children age 5 to 11 years old from low socio-economic areas in England and reported a GM of 0.37 ng/ml [27], while more recent data after the implementation of national smoke-free restrictions in the UK have produced GM salivary cotinine concentrations in 10-11 year old school children of 0.22 ng/ml in Scotland [13] and of 0.15 ng/ml in Wales [14]. Willers and colleagues measured cotinine concentrations among children aged 8 to 13 years old in Sweden and found median concentrations of 0.62 ng/ml among children who presented with asthmatic symptoms [28] lower than the median salivary cotinine value in our study (0.72 ng/ml).

Table 6 presents an overview of selected relevant studies looking at cotinine concentrations among children living in smoking and non-smoking homes globally. The eight studies selected have either performed cotinine assays on serum or salivary samples. The ages of the children were from less than 5 years old to 19 years old and the sample size varies from 159 to 13365 children. Serum cotinine concentrations from three studies found that a GM value of 0.03 and 0.10 ng/ml among children living in non-smoking homes [29-31]. The GM salivary cotinines for children living in smoking homes were higher at 0.60, 0.84 and 1.05 ng/ml respectively [29-31]. The study by Dove et al. [29] represents GM cotinine concentration for children living in USA counties with extensive SFL being practiced, thus the low GM serum cotinine concentrations compared to the other two studies. Five studies report salivary cotinine data with the lowest GM cotinine concentration was among children living in non-smoking homes in the Scottish study (0.07 ng/ml) [13]. Results from two other English studies [32,33] indicate GM salivary cotinine concentrations of 0.22 and 0.32 ng/ml for children living in non-smoking homes compared to the 0.32 ng/ml observed in this study. In other studies of children living with at least one smoker, GM salivary cotinine concentrations of 0.84 ng/ml [32], 0.38 [27], 0.37 [33] and 0.32 ng/ml [13] have been reported.

**Table 6:** An overview of selected studies looking at cotinine concentrations among children and youth globally

Authors and country	Type of study and cotinine sample	Size of population	Information of study	GM cotinine concentrations
**Serum cotinine**				
Lazcano-Ponce et al. 2007 Mexico [31]	National Health Survey year 2000	76 and 83 children (<5 years)	Non-smoking homes and smoking homes	0.10 and 0.60 ng/ml for non-smoking and smoking homes respectively
Dove et al. 2010 USA [29]	National Health and Nutrition Examination Survey 1999-2006	11 486 non-smoking children/youth (<19 years)	Divided into 3 groups of extensive, limited and no SFL coverage	0.03 and 0.84 ng/ml for non-smoking and smoking homes in county with extensive SFL, 0.05 and 0.90 ng/ml for limited SFL, 0.07 and 1.13 ng/ml for no SFL coverage
Marano et al. 2009 USA [30]	National Health and Nutrition Examination Survey 2003-2006	5518 children/adolescents (3-19)	Non-smoking homes and smoking homes	0.05 and 1.05 ng/ml for non-smoking and smoking homes respectively
**Salivary cotinine**				
Akhtar et al. 2007 Scotland [13]	National Survey from 111 schools	2559 and 2424 children (11 years) surveyed in 2006 and 2007 respectively	Children represented exposure before and after the implementation of SFL	0.14 and 0.07 ng/ml for children living in non-smoking homes before and after SFL 0.57 and 0.32 ng/ml for children living in smoking homes (father only smokes) after the SFL
Whitrow et al. 2010 England [32]	Survey in 2003-2004 among children from 51 schools in London	2311 children (11-13 years)	To differentiate exposure of SHS among whites and other ethnic groups	0.30 ng/ml and 0.84 ng/ml among White children living with no smokers and smokers (father only smokes) respectively
Jarvis et al. 2009 England [33]	Survey among children between year 1996 to 2007	13365 children (4-15 years)	Relationship between cotinine and smoking restriction in homes	0.22 ng/ml for non-smoking homes, 0.37 ng/ml (one smoker) and 0.71 ng/ml (two smokers) for smoke-free homes 1.67 ng/ml (one smoker) and 2.46 ng/ml (two smokers) for homes with no smoking restrictions
^a ^Delpisheh et al. 2007 England [27]	Survey among children in low socioeconomic area in 2004	425 children (5-11 years)	Relationship between cotinine and respiratory symptoms	0.37 ng/ml for all children, 0.56 ng/ml among children living with a smoking mother and 0.38 ng/ml among children living with a smoking father
Holliday et al. 2009 Wales [14]	National Survey from 75 schools	1750 children (10-11)	Children represented exposure before and after the implementation of SFL	0.17 ng.ml for all children before the SFL 0.15 ng/ml for all children after the SFL

^a ^No specific value available for children living in non-smoking homes

Figure 1 illustrates the distribution of Malaysian children's salivary cotinine levels as compared to two important and relevant levels derived from two separate studies [25,26]. The 1.7 ng/ml level has been implicated as a level where cardiovascular changes can be observed in children, while the 3.0 ng/ml figure reflects the average value found in heavily SHS-exposed non-smoking bar workers in Scotland prior to smoke-free legislation. From the findings of this study, 1 in 5 schoolchildren have cotinine concentrations at or above the 1.7 ng/ml threshold. The present study also found that approximately 1 in 20 Malaysian schoolchildren have salivary cotinine concentrations at or above the 3 ng/ml level. It is of concern that approximately 5% of 10-11 year olds in Malaysia may be experiencing SHS exposures of similar duration and intensity to those experienced by hospitality workers prior to smoke-free restrictions.

Children living in non-smoking homes in Malaysia (0.32 ng/ml) have GM salivary cotinine levels nearly 5 times higher than those of Scottish children (0.07 ng/ml) [13]. This finding is suggestive of the fact that the partial nature of smoking restrictions practiced in Malaysia leads to considerable non-home exposure when compared to Scottish children. Exposures of SHS among children living with non-smoking parents have been shown to be reduced where comprehensive smoke-free legislation is introduced [13]. Smoke-free legislation can play an important role in reducing the intensity and duration of children's exposure to SHS in settings outside of the home.

In the multiple regression analysis, one of the strongest predictor of children's GM salivary cotinine concentration was having a father who smoked. The skewed gender distribution among parents in this study confirms what is already known about the smoking characteristics of adults in Malaysia [10]. This study however reports a lower prevalence of maternal smoking (0.3%) compared to the Malaysian National Health Morbidity Survey III (1.6%) [34].

Household smoking 'rules' or restrictions have commonly been used in other studies as a means of assigning potential exposures [33,35]. Children living in homes with total smoking restrictions had GM cotinine concentrations that were approximately two-thirds of children living in homes with no smoking restrictions (0.45 v 0.74 ng/ml). There is a greater difference in rural homes than urban homes, suggesting more outside home exposure to SHS in urban children. From the multiple linear regression living in the urban areas was one of the strongest influences on the levels of salivary cotinine concentrations in children. We do not know why this may be but some possibilities include the possibility that urban-living parents smoke more and/or the fact that urban children have more outside home exposure to SHS during transport to school or within cafes and restaurants commonly available in urban areas.

This study reports disagreement between children's and parental report of exposure hours to SHS. The differences observed for the exposure hours in this study could be explained by parental awareness of negative implications associated with SHS exposure which may have lead to the under-reporting of exposures, especially among parent who are smokers themselves. One study have mentioned that parental-reported SHS exposures may be unable to identify exposures occurring outside of home during time spent away from the parent [36]. This study indicates that parental report used in many studies looking at children's exposure to SHS smoke may underreport actual exposure to SHS and this point should be considered in future epidemiological studies [37,38].

## Limitations of study

The schools selected in this study under-represent children of Chinese and Indian backgrounds. As the Malay population has been reported to have the highest prevalence of current smokers (28.9%) compared to the Chinese (18.7%) and Indians (16.8%) in Malaysia, it is likely that the findings of this study over-estimate the exposure of Malaysian schoolchildren at a national level [10]. Additionally, cotinine concentrations were found not to be significantly associated with family earnings unlike studies in the UK [13,27]. It is acknowledged that a larger portion of the respondents in this study comes from families categorised within the lower socio-economic levels according to the self-report of family income data however the differences were not significant. The participants in this study are over represented by schoolchildren coming from low to middle socio-economic levels. The percentage of non-smoking households observed among participants who were not involved in providing saliva samples were found to be higher than the participants who were involved in providing the saliva samples. The percentage of non-participating children who lived in non-smoking households was 56.5% compared to 47.1% of participating schoolchildren. Schoolchildren who provided saliva samples in this study were thus more likely to come from households with smokers and so again it is more likely that this study may over estimate cotinine concentrations of the wider population of Malaysian schoolchildren. For these identified reasons, it is important that the results from this study are interpreted with care and not generalised to the whole of the Malaysian population.

Children's smoking status was defined as non-smoker on the basis of their salivary cotinine levels being less than 15 ng/ml. Our study did not enquire about the smoking status of children directly as this would have been culturally sensitive. However, we note that only 2 out of 1064 schoolchildren have cotinine concentrations above 10 ng/ml and only 15 out of 1064 have cotinine concentrations above 5 ng/ml. Thus, we do not think smoking was common among this population.

## Conclusions

The findings of this study provide new and important information on salivary cotinine concentrations among schoolchildren in Malaysia. Over half of schoolchildren in Malaysia live with one or more adult smokers. The GM of salivary cotinine concentrations of Malaysian schoolchildren after the implementation of smoke-free legislation in Malaysia is 0.46 ng/ml and is significantly higher among schoolchildren living with smokers at home when compared to children living with non-smokers.

Overall, Malaysian schoolchildren living in non-smoking households have higher salivary cotinine concentrations than children of similar ages in other countries, suggesting a significant amount of Malaysian children's SHS exposure occurs outside the home. The partial nature of smoking restrictions in semi-enclosed public areas in Malaysia may be one possible explanation for this. More research is required to identify the sources of exposure of Malaysian children to SHS. There is a need to examine the proportion of children's SHS exposure that arises during home life and that from time spent in public places. Given that about 5% of Malaysian children have salivary cotinine values comparable to heavily SHS-exposed bar workers there is also clearly a need for policies and interventions to reduce children's SHS exposure in Malaysia. From the findings of this study, it is legitimate to consider whether current Malaysian smoke-free legislation is sufficient to protect children from SHS exposure in public spaces.

## Competing interests

The authors declare that they have no competing interests.

## Authors' contributions

EZA carried out the data collection, performed the statistical analysis and drafted the manuscript. AO carried out the salivary cotinine assays. SS and JGA conceived the study, participated in its design and helped to draft the manuscript. HAR helped in the design of the study and helped to draft the manuscript. SWT helped draft the manuscript. All authors read and approved the final manuscript.

## Authors' information

EZA is a PhD student in the field of Environmental Medicine at the University of Aberdeen. SS is a senior lecturer based in the Environmental and Occupational Medicine group at the University of Aberdeen. AO is a biochemist in the Institute of Medical Research, Malaysia. HAR is an associate professor at the Department of Community Health, Faculty of Medicine and Health Science, University Putra Malaysia. SWT is a paediatrician and senior lecturer at the Department of Child Health, University of Aberdeen. JGA is the director of the Institute of Occupational Medicine, University of Birmingham.

## Pre-publication history

The pre-publication history for this paper can be accessed here:

http://www.biomedcentral.com/1471-2458/11/634/prepub
